# Enhanced Expression of ABCB1 and Nrf2 in CD133-Positive Cancer Stem Cells Associates with Doxorubicin Resistance

**DOI:** 10.1155/2020/8868849

**Published:** 2020-08-12

**Authors:** Shinji Goto, Tsuyoshi Kawabata, Tao-Sheng Li

**Affiliations:** Department of Stem Cell Biology, Atomic Bomb Disease Institute, Nagasaki University, Nagasaki 852-8523, Japan

## Abstract

The precise mechanism about drug resistance of cancer stem cells (CSCs) has not yet been completely understood. Based on the expression of CD44 and CD133, two well-recognized cell surface markers for CSC identification, we tried to separate HCT8 colorectal cancer cells into different subpopulations and then investigated how the expression of CD44 and CD133 associated with doxorubicin (DXR) resistance. Interestingly, DXR resistance was observed in CD44^+^CD133^+^ (*P* < 0.01*vs.* all other subpopulations), but not in CD44^+^CD133^−^ cells. CD44^+^CD133^+^ cells also showed an enhanced expression of ABCB1 and drug efflux ability (*P* < 0.001*vs.* all other subpopulations), but verapamil, an inhibitor of ABCB1, only partially mitigated the DXR resistance. Independent on the accumulation of DXR, lower level of reactive oxygen species and higher expression of Nrf2 were detected in CD44^+^CD133^+^ than CD44^+^CD133^−^ cells (*P* < 0.05). Unexpectedly, silencing CD133 by siRNA only partially enhanced the cytotoxicity of DXR, but did not obviously change the expression of ABCB1 and the accumulation of DXR in CD44^+^CD133^+^ cells. Complex mechanisms, including drug excretion and redox regulation, are likely involved in the DXR resistance of CD133-positive cells, suggesting the difficulty of drug resistance problem in cancer chemotherapy.

## 1. Introduction

The heterogeneity of cancer cells is generally accepted, and a stem cell-like subpopulation that is called “cancer stem cells” (CSCs) has been identified in various types of malignant tumors. Although the lack of consensus on the definition, CSCs are widely recognized as a small subpopulation among cancer cells with the properties of self-renewal and tumor initiation. As CSCs play a critical role in the recurrence and metastasis of cancer [1], targeting the CSCs is thought to be a promising approach for curing cancer.

A large number of past studies have tried to identify and characterize the CSCs. As normal tissue-specific stem cells are considered as the main origin of cancer [2], the CSCs are also thought to be inherited, at least partially, the characterization of normal tissue-specific stem cells. Therefore, many studies on the identification/purification of CSCs have simply shared markers of hematopoietic stem cells, including the most popularly used cell surface markers of CD44 and CD133 [3, 4]. CD44 is a type I transmembrane glycoprotein that is expressed on hematopoietic, fibroblastic, and glial cells and functionally known to mediate cell-cell and cell-matrix interactions. Previous studies have demonstrated that the CD44 is not only a biomarker but also plays critical roles in the maintenance of CSCs, the resistance to various therapies/stresses, and the metastasis of cancer cells [5–11]. CD133 is originally identified as protein expressing on the cell surface of hematopoietic stem cells [12] and has subsequently been found to be critical in the maintenance of “stemness” of stem cells in various tissues [13–18]. CD133 has also been found in some CSC [19–22], which contributes to therapeutic resistance through the activation of Akt, Bcl-2, and MAPK/PI3K signaling pathways [23–26]. Although the expressions of CD44 and CD133 in cancer cells likely associate with the resistances to radiotherapy, chemotherapy, and various stresses, the different significance between CD44 and CD133 has not yet been well understood.

In this study, we investigated whether the expression of CD44 and CD133 in human colorectal cancer cells (HCT8) differently contributed to drug resistance. Our data indicated that the expression of CD133, rather than CD44, closely associated with doxorubicin (DXR) resistance, at least partially through drug excretion and redox regulation.

## 2. Materials and Methods

### 2.1. Cell Culture

Human colorectal cancer (HCT8) cells were cultured in RPMI 1640 medium (FUJIFILM Wako Pure Chemical, Japan) supplemented with 10% FBS (GIBCO, Thermo Fisher Scientific, MA, USA) at 37°C, in a humidified atmosphere of 95% air and 5% CO_2_.

### 2.2. Separation of CD44- and CD133-Positive Cells from HCT8 Cells

We separated the parent HCT8 cells into CD44-positive (CD44^+^) and CD133-positive (CD133^+^) cells by a two-step magnetic cell sorting method as described previously [13, 27]. Briefly, HCT8 cells were collected as a single-cell suspension by trypsinization and then incubated with magnetic microbead-conjugated anti-human CD44 antibody (Miltenyi Biotec, Germany) for 30 min. After washing, cells were separated into CD44^−^ and CD44^+^ subpopulations by using the autoMACS™ Pro separator (Miltenyi Biotec), according to the manufacturer's instruction. The purified CD44^+^ cells were further expanded and then harvested as a single-cell suspension to be incubated with magnetic microbead-conjugated anti-human CD133 antibody (Miltenyi Biotec) for 30 min. After washing, the CD44^+^CD133^−^ and CD44^+^CD133^+^ subpopulations were separated as described above. This two-step isolation enabled us to obtain a sufficient number of CD44^−^, CD44^+^, CD44^+^CD133^−^, and CD44^+^CD133^+^ cells for our experiments.

To verify the purity of each subpopulation, isolated cells were stained with PE-labelled mouse anti-human CD133 (clone: AC133) (Miltenyi Biotec) and FITC-labelled mouse anti-human CD44 (clone: DB105) (Miltenyi Biotec), according to the supplied protocols. Flow cytometry analysis was performed using a FACSCalibur (Becton Dickinson), as described previously [27]. Mouse IgG1-PE (Miltenyi Biotec) and mouse IgG1-FITC (Miltenyi Biotec) were used as a negative control.

### 2.3. Cytotoxicity Assays

Cells were seeded in 96-well culture plates at a density of 2 × 10^4^ cells per well and cultured overnight. The cells were then treated with various concentrations of DXR (FUJIFILM Wako Pure Chemical), in the absence or presence of verapamil (FUJIFILM Wako Pure Chemical). Cytotoxicity assays were performed using the Cell Proliferation Kit I (MTT) (Roche Applied Science, Germany), as described previously [27]. The absorbance was measured at 570 nm using a microplate reader (Multiskan FC, Thermo Fisher Scientific).

### 2.4. Analysis on the Expression of ABC Transporters

The expressions of the ATP-binding cassette subfamilies of B member 1 (ABCB1) or G member 2 (ABCG2) were analyzed by flow cytometry. Briefly, cells were incubated with mouse primary antibodies against human ABCB1 and ABCG2 (BD Biosciences, CA, USA) and then labeled by FITC-conjugated anti-mouse IgG (BD Biosciences), according to the manufacturer's instruction. Respective isotype controls were used as a negative control. After washing, flow cytometry analysis was performed using a FACSCalibur.

### 2.5. Analysis of Cellular Accumulation of DXR

The intracellular accumulation of DXR was analyzed by flow cytometry. Briefly, cells were treated by 10 *μ*M DXR for 24 hr, in the absence or presence of 50 *μ*M verapamil or 200 *μ*M buthionine sulfoximine (BSO, Sigma-Aldrich, MO, USA). Cells were then collected as a single-cell suspension and washed twice with ice-cold phosphate-buffered saline. The accumulation of DXR within cells was evaluated by the intracellular fluorescence intensity, using a FACSCalibur. The nucleus accumulation of DXR was analyzed by using cell pellets treated with 0.1% Triton X-100/PBS as assay material, as described previously [28].

### 2.6. Detection of Intracellular ROS

The intracellular ROS level based on the oxidation of 2′,7′-dichlorodihydrofluorescein diacetate (H_2_DCFDA, Molecular Probes, Thermo Fisher Scientific) was measured to form the fluorescent compound 2′,7′-dichlorofluorescein (DCF), using a FACSCalibur.

### 2.7. Immunoblot Analysis

Expression levels of phosphorylated-p38 MAP kinase (phospho-p38MAPK), total p38MAPK, and nuclear factor erythroid 2-related factor 2 (Nrf2) in the cells were estimated by immunoblotting. Briefly, cell lysate (30 *μ*g of total protein) was separated by sodium dodecyl sulfate–polyacrylamide gel electrophoresis (SDS-PAGE) gel, transferred to PVDF membranes (Bio-Rad, CA, USA), and then incubated with primary antibodies (Cell Signaling Technology, MA, USA), followed by appropriate HRP-labeled secondary antibodies (DAKO, Agilent Pathology Solutions, CA, USA). Blots were developed by enhanced chemiluminescence, using an ECL kit (GE Healthcare Life Sciences, PA, USA). Semiquantitation was done by measuring the density of bands, using the Image Quant LAS 4000 Mini biomolecular imager (GE Healthcare Life Sciences), as described previously [27].

### 2.8. siRNA Treatment

Small interfering RNA- (siRNA-) specific targeting to CD133 (On TARGETplus siRNA) and a scramble siRNA (On TARGETplus siRNA negative control) were obtained from Dharmacon (Horizon Discovery, Cambridge, UK). Cells were seeded in 6-well plates (2 × 10^5^ cells/well) and incubated for 16 hr. Transfections were performed using DharmaFECT 1 siRNA Transfection Reagents (Dharmacon), according to the manufacturer's instructions. Analyses were done at 48 hr after siRNA transfection.

### 2.9. Statistical Analysis

All of the results are presented as the means ± S.D. Statistical significance was determined by one-way analysis of variance (ANOVA) followed by Tukey's test (Dr. SPSS II, Chicago, IL). Differences were considered significant when *P* < 0.05.

## 3. Results

### 3.1. HCT8 Cells Were Separated into Various Subpopulations Based on Their Expressions of CD44 and CD133

**Figure 1:**
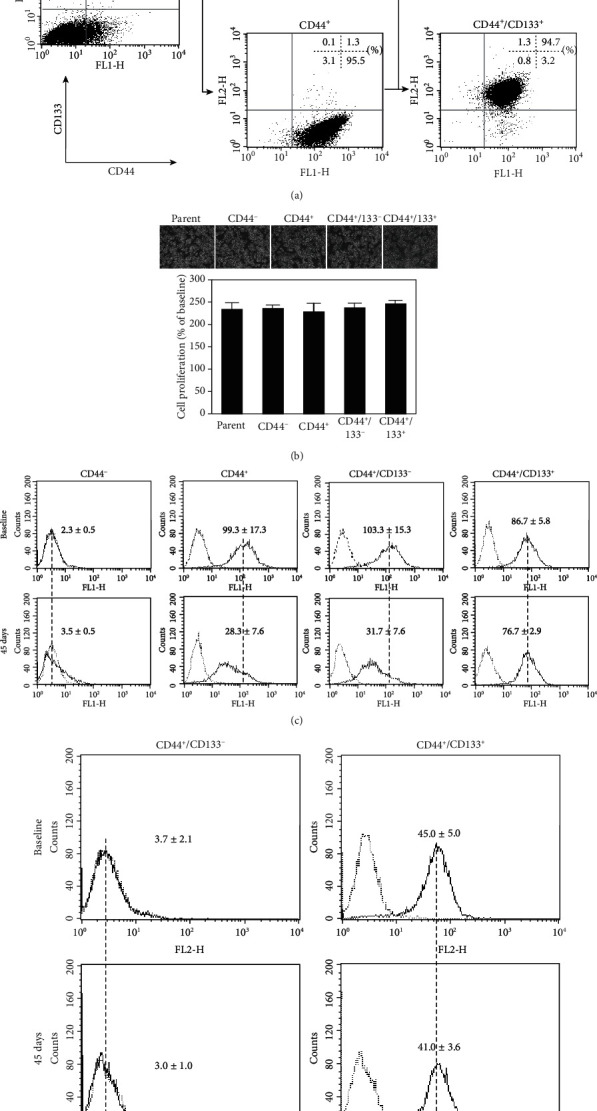
The separation of HCT8 colorectal cancer cells into different subpopulations based on the expression of CD44 and CD133. (a) Representative dot plots of flow cytometry analysis show the purities of each subpopulation of isolated cells. Quantitative data in the dot plots are presented as the percentages of positive cells from three independent experiments. (b) Representative photos of morphological properties (upper) and MTT assay on cell growth (lower) at 24 hr after the initiation of culture. Data are presented as the mean ± SD from three independent experiments. (c, d) Representative histograms of flow cytometry analysis showed the expressions of CD44 (c) and CD133 (d) at baseline and 45 days after cell culture. The dotted vertical lines through histograms indicate the difference in the expression peaks between the baseline and at 45 days after culture. Quantitative data in the histograms are presented as the mean fluorescent intensity from three independent experiments.

First, we separated the HCT8 cells into CD44^−^ and CD44^+^ subpopulations and compared their sensitivity to anticancer drugs of DXR and cisplatin (cis-diaminedichloroplatine, CDDP). However, no difference in the sensitivity to the two drugs was observed between CD44^+^ and CD44^−^ cells (data not shown). We further tried to purify a small population of CD133^+^ cells from these CD44^+^ cells (CD44^−^ cells almost negatively expressed with CD133, Figure 1(a)). As a result, we separated HCT 8 cells into different subpopulations, including CD44^−^, CD44^+^, CD44^+^CD133^−^, and CD44^+^CD133^+^ cells. The purities of isolated cells in each subpopulation were confirmed to be around 95% by flow cytometry (Figure 1(a)).

### 3.2. Growth and Phenotype Change in Different Subpopulations of Cells

The morphology and proliferation of these cells could not be found obviously different among subpopulations (Figure 1(b)). The expression of CD44 in all subpopulations kept stable within 30 days of reculturing from the frozen cells that stocked immediately after isolation. Interestingly, the expression of CD44 was a tendency to decrease with culture time in CD44^+^ (fluorescence intensity: 99.3 ± 17.3 at baseline *vs.*28.3 ± 7.6 at 45 days, *P* < 0.001; Figure 1(c)) and CD44^+^CD133^−^ cells (fluorescence intensity: 103.3 ± 15.3 at baseline *vs.*31.7 ± 7.6 at 45 days, *P* < 0.001; Figure 1(c)) but still kept stable in CD44^+^CD133^+^ cells at 45 days after reculturing (fluorescence intensity: 86.7 ± 5.8 at baseline *vs.*76.7 ± 2.9 at 45 days, *P* = 0.84; Figure 1(c)). The expression of CD133 in CD44^+^CD133^+^ cells kept very stable (fluorescence intensity: 45.0 ± 5.0 at baseline *vs.*41.0 ± 3.6 at 45 days, *P* = 0.49; Figure 1(d)), and CD44^+^CD133^−^ cells did not turn to express CD133 within 45 days of reculturing (fluorescence intensity: 3.7 ± 2.1 at baseline *vs.*3.0 ± 1.0 at 45 days, *P* = 0.99; Figure 1(d)). Therefore, we used the cells within 30 days after reculturing from the frozen stocked cells in subsequent experiments.

### 3.3. DXR Resistance of CD44^+^CD133^+^ Cells

**Figure 2:**
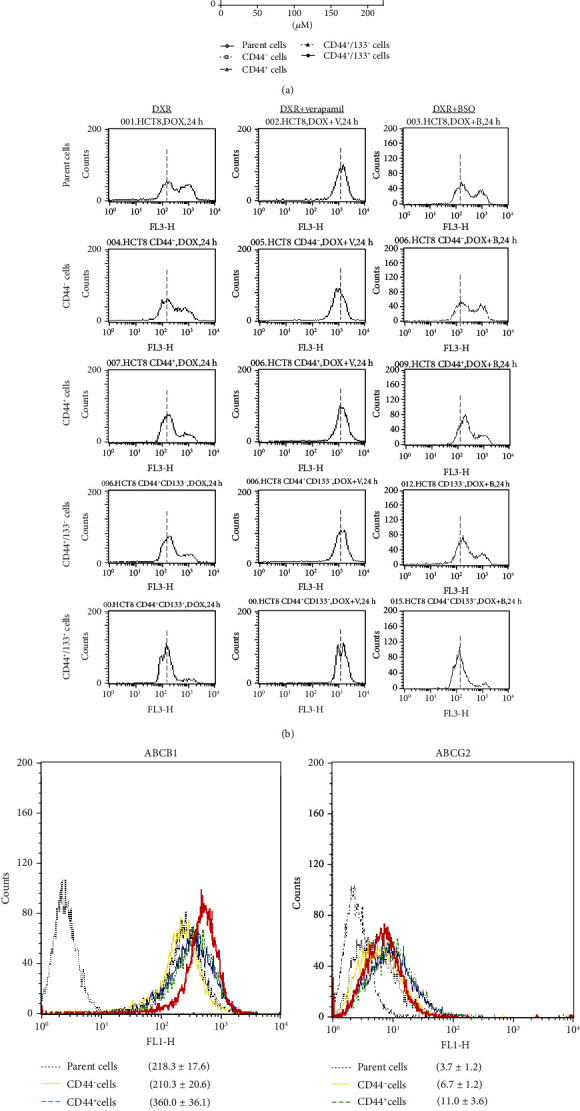
DXR resistance of different subpopulations of cells. (a) MTT assay was done to evaluate the cytotoxicity of DXR. Data are expressed as the percentile of baseline (before DXR treatment) from three independent experiments. ^∗^*P* < 0.01 vs. all other subpopulations. (b) Representative histograms of flow cytometry analysis show the accumulation of DXR in cells 24 hr after the treatment with 10 *μ*M DXR, in the absence or presence of 50 *μ*M verapamil and 200 *μ*M BSO. The dotted vertical lines through histograms indicated the mean levels of DXR accumulation in CD44^+^CD133^+^ cells for comparing with other subpopulations of cells. The results were reproducible in three independent experiments. (c) Representative histograms of flow cytometry analysis show the expression of the ABCB1 or ABCG2 in different subpopulations of cells. Quantitative data in the histograms are presented as the mean fluorescent intensity from three independent experiments.

Next, we evaluated the sensitivity of cells to DXR by MTT assay. With the addition of 10~200 *μ*M of DXR in medium, we found that the survival of CD44^+^CD133^+^ cells was significantly higher than all other subpopulations of cells after 48 hr of culture (*P* < 0.01*vs.* other groups at different DXR concentrations, Figure2(a)).

To understand the relevant mechanism, we measured the intracellular accumulation of DXR in cells by flow cytometry. The accumulation of DXR in CD44^+^CD133^+^ cells was detected as the lowest among these subpopulations, at 24 hr after the exposure to 10 *μ*M DXR (Figure 2(b)). We further found that the intracellular accumulation of DXR in CD44^+^CD133^+^ cells was obviously increased by the treatment with verapamil, an inhibitor for drug efflux cell membrane transporters of ABCB1 and ABCG2 (Figure 2(b)). However, the intracellular accumulation of DXR in CD44^+^CD133^+^ cells did not change by the treatment with BSO, a glutathione synthesis inhibitor that indirectly regulates drug efflux through ABCC1 (Figure 2(b)). We also confirmed that the expression of ABCB1 (*P* < 0.01*vs.* other groups), but not ABCG2, was enhanced in CD44^+^CD133^+^ cells (Figure 2(c)), suggesting the probable role of ABCB1 on DXR resistance in CD44^+^CD133^+^ cells.

**Figure 3:**
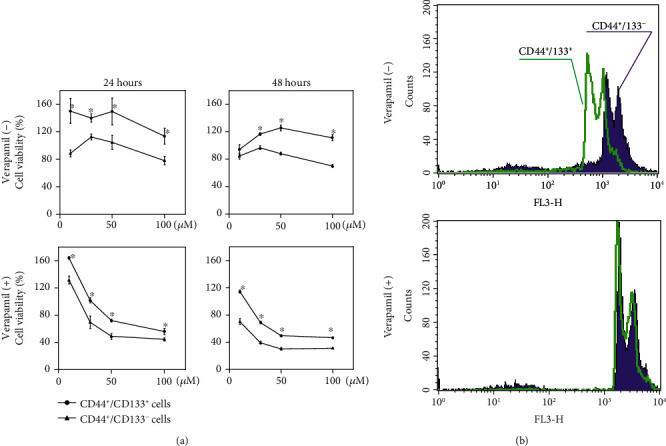
DXR resistance and nuclear DXR accumulation in CD44^+^CD133^−^ and CD44^+^CD133^+^ cells, in the absence or presence of drug efflux inhibitor. (a) MTT assay was done to compare the cytotoxicity of DXR in CD44^+^CD133^−^ and CD44^+^CD133^+^ cells, with or without the addition of 50 *μ*M verapamil. Data were expressed as a percent of baseline (before DXR treatment) from three independent experiments. ^∗^*P* < 0.05 vs. CD44^+^CD133^−^ cells. (b) Representative histograms of flow cytometry analysis showed the nuclear accumulation of DXR in cells 24 hr after the treatment by 10 *μ*M DXR, with or without the addition of 50 *μ*M verapamil. The results were reproducible in three independent experiments.

To further confirm the causal relationship between the enhanced drug efflux and DXR resistance, we evaluated the cytotoxicity of DXR, in the presence or absence of verapamil. Unexpectedly, verapamil only partially enhanced the cytotoxicity of DXR, in either CD44^+^CD133^+^ or CD44^+^CD133^−^ cells (Figure 3(a)).

It is well known that DXR interacts with nuclear DNA to inhibit macromolecular biosynthesis. Therefore, we also estimated the effect of verapamil on the nuclear accumulation of DXR. The nuclear accumulation of DXR was observed obviously less in CD44^+^CD133^+^ than CD44^+^CD133^−^ cells but tended to have comparable levels with verapamil treatment (Figure 3(b)).

### 3.4. CD44^+^CD133^+^ Cells Showed Better Stress Tolerance than CD44^+^CD133^−^ Cells

**Figure 4:**
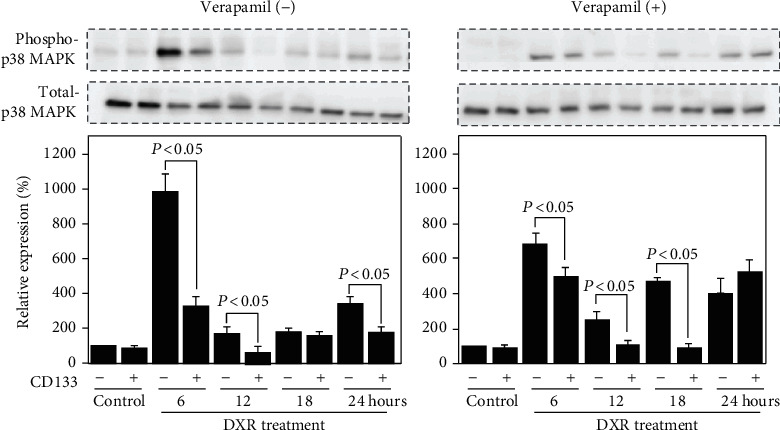
Different expression of phosphorylated p38MAPK between CD44^+^CD133^−^ and CD44^+^CD133^+^ cells. Representative blots and semiquantitative data on the expression of phosphorylated p38MAPK and total p38MAPK in cells treated with 10 *μ*M DXR, in the absence or presence of 50 *μ*M verapamil. The quantitative data are normalized to total p38MAPK. Data are expressed as relative values to CD44^+^CD133^−^ cells without DXR treatment and presented as the mean ± SD from three independent experiments.

It is well known that the stress response kinase p38MAPK can be activated by various extracellular stresses and plays critical roles in cell survival and apoptosis. Although the basal level of phosphorylated p38MAPK was detected very similar between CD44^+^CD133^+^ and CD44^+^CD133^−^ cells (*P* = 0.92, Figure 4), lower expression was observed in CD44^+^CD133^+^ than CD44^+^CD133^−^ cells after DXR exposure, even under verapamil treatment (*P* < 0.05, Figure 4). This suggests a better tolerance to stress of CD44^+^CD133^+^ cells, independent on the accumulation of DXR.

### 3.5. CD44^+^CD133^+^ Cells Showed Higher Antioxidant Capacity than CD44^+^CD133^−^ Cells

**Figure 5:**
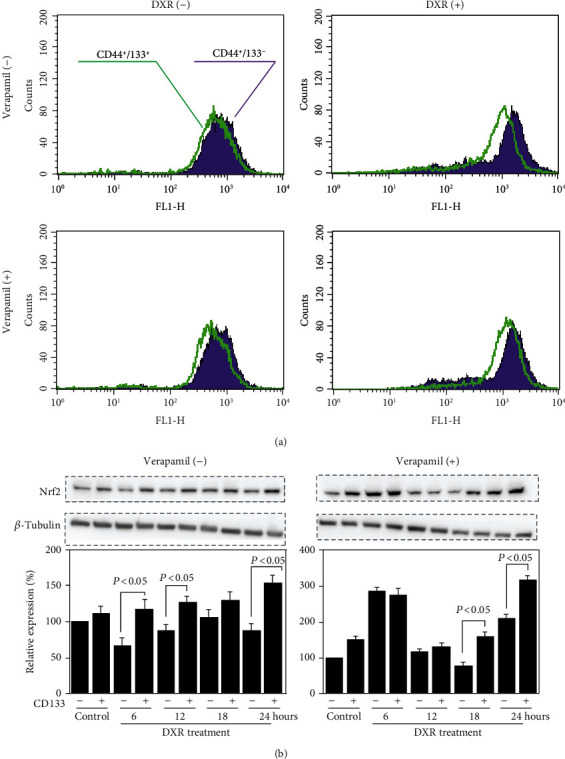
Different antioxidant capacity between CD44^+^CD133^−^ and CD44^+^CD133^+^ cells. (a) Representative histograms of flow cytometry analysis show the intracellular ROS levels 24 hr after the treatment by 10 *μ*M DXR, in the absence or presence of 50 *μ*M verapamil. The results were reproducible in three independent experiments. (b) Representative blots and semiquantitative data on the expression of Nrf2 in cells treated with 10 *μ*M DXR, in the absence or presence of 50 *μ*M verapamil. The quantitative data are normalized to *β*-tubulin. Data are expressed as relative values to CD44^+^CD133^−^ cells without DXR treatment and presented as the mean ± SD from three independent experiments.

It is also well known that DXR generates ROS, and oxidative stress due to ROS generation may induce the activation of p38MAPK. Therefore, we estimated the ROS levels in cells, with or without DXR exposure. We observed a lower level of ROS in CD44^+^CD133^+^ than CD44^+^CD133^−^ cells, especially under DXR exposure, but verapamil did not obviously change the ROS levels (Figure 5(a)). Based on these findings, we speculated that the enhanced antioxidant capacity in CD44^+^CD133^+^ cells might help to maintain a lower level of phosphorylated p38MAPK.

Nrf2, a transcription factor that is well known to be activated by oxidative stress, such as ROS and electrophilic substances, can protect cells against various stresses. We also compared the expression level of Nrf2 between CD44^+^CD133^+^ and CD44^+^CD133^−^ cells. Western blotting showed a higher expression of Nrf2 in CD44^+^CD133^+^ than CD44^+^CD133^−^ cells, especially under DXR exposure (*P* < 0.05, Figure 5(b)), and the enhanced expression of Nrf2 in CD44^+^CD133^+^ cells was not cancelled by verapamil treatment (*P* < 0.05, Figure 5(b)).

### 3.6. siRNA Treatment

**Figure 6:**
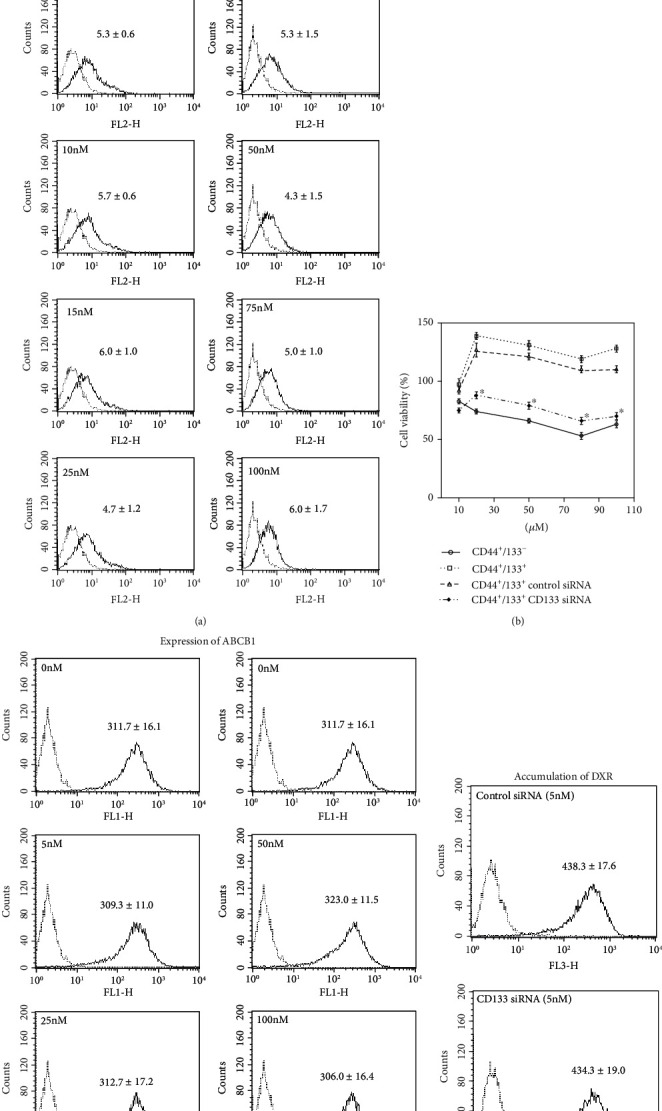
The effect of silencing CD133 expression on DXR resistance of CD44^+^CD133^+^ cells. (a) Representative histograms of flow cytometry analysis on the expression of CD133 in CD44^+^CD133^+^ cells after silencing by different dosages of targeted siRNA. Quantitative data in the histograms are presented as the mean fluorescent intensity from three independent experiments. (b) MTT assay was done to evaluate the cytotoxicity to DXR. Cells were treated with 5 nM siRNA for 48 hr followed by DXR treatment for another 48 hr. Data are expressed as a percent of baseline (before DXR treatment) from three independent experiments. ^∗^*P* < 0.05 vs. CD44^+^CD133^−^ cells. (c) Representative histograms of flow cytometry analysis on the expression of ABCB1 in cells after silencing by different dosages of targeted siRNA. Quantitative data in the histograms are presented as the mean fluorescent intensity from three independent experiments. (d) Representative histograms of flow cytometry analysis on the accumulation of DXR. Quantitative data in the histograms are presented as the mean fluorescent intensity from three independent experiments.

To further confirm the regulatory role of CD133 in drug resistance, we tried to silence CD133 expression in CD44^+^CD133^+^ cells by siRNA and then estimated cytotoxicity of DXR. Although the decrease of CD133 expression was clearly observed by targeted siRNA (*P* < 0.001*vs.* 0 nM, Figure 6(a)), DXR resistance of CD44^+^CD133^+^ cells only partially improved (Figure 6(b)). Unexpectedly, the silencing of CD133 did not change the expression of ABCB1 in CD44^+^CD133^+^ cells, even using excessive concentrations of CD133 siRNA (*P* = 0.89*vs.* 0 nM, Figure 6(c)). We also confirmed that the silencing of CD133 did not affect the accumulation of DXR in CD44^+^CD133^+^ cells (*P* = 0.98*vs.* control siRNA, Figure 6(d)).

This suggests that, beyond the drug excretion and redox regulation, other complex mechanisms are also likely involved in the DXR resistance in CD44^+^CD133^+^ cells.

## 4. Discussion

By using the well-recognized cell surface markers of CD44 and CD133 for CSC identification, we tried to separate the HCT8 human colon cancer cells into CD44^−^, CD44^+^, CD44^+^CD133^−^, and CD44^+^CD133^+^ subpopulations and then investigated how the expressions of CD44 and CD133 associated with drug resistance. Actually, we checked several cancer cell lines on the expression of CD44 and CD133, including HeLa cells and A549 cells. However, both HeLa cells and A549 cells showed almost 100% expression of CD44. Only the HCT8 cells showed a partial expression of CD44 (about 30%) and a rare expression of CD133. Therefore, we only isolated different subpopulations from HCT8 cells for this study.

First, we found that the expression level of CD44 kept very stable in the CD44^+^CD133^+^ cells but gradually declined in CD44^+^CD133^−^ cells during a cell passaging process. On the other hand, some of CD44^−^ cells shifted to express CD44 during a cell passaging process (Figure 1(c)). These findings suggested the plasticity of CD44 expression in HCT8 cells. Actually, Ohata et al. reported that CD44 high-expressed cells from human intractable colon cancer patients can differentiate into CD44 low-expressed cells, and a fraction of CD44 low-expressed cells can also generate CD44 high-expressed cells in a xenograft mouse model [29]. However, it is unclear why the CD44^+^CD133^+^ cells, but not CD44^+^CD133^−^ cells, stably maintain the expression level of CD44. Unlike the extensive expression of CD44 with high plasticity, the expression of CD133 was only observed in very few of the HCT8 cells with poor plasticity.

A number of previous studies have demonstrated that CSCs are likely resistant to chemotherapeutic drugs. The CD44^+^CD133^+^ cells, but not the CD44^+^ and CD44^+^CD133^−^ cells, showed DXR resistance (Figure 2(a)). According to this data, the expression of CD133, but not CD44, seems to be closely associated with drug resistance. Actually, these CD44^+^CD133^+^ cells showed the enhanced expression of ABCB1 and the decreased intracellular accumulation of DXR (Figures 2(b) and 2(c)). Liu et al. reported that non-small-cell lung cancer cells treated with low-dose CDDP are sufficient to enrich CD133^+^ cells and upregulate ABCB1 expression through Notch signaling, which therefore increases the cross-resistance to DXR [30]. However, the inhibition of ABCB1 by verapamil only partially improved the DXR resistance of CD44^+^CD133^+^ cells in this study.

To find other potential mechanisms involving in the DXR resistance of CD44^+^CD133^+^ cells, we investigated several interesting aspects, including the stress protection and redox regulation. We found that p38MAPK, one of the most popular protein kinases known to be activated by inflammatory cytokines, lipopolysaccharide, osmotic shock, ultraviolet light, and other stresses, was more obviously induced by DXR in CD44^+^CD133^−^ cells than CD44^+^CD133^+^ cells (Figure 4). Moreover, the activation of p38 MAPK was not dependent on the intracellular accumulation of DXR (Figure 4).

DXR is known to insert between the base pairs of DNA of tumor cells and exhibits antitumor effects by suppressing the biosynthesis of both DNA and RNA through the inhibition of DNA polymerase, RNA polymerase, and topoisomerase II reactions. Furthermore, it is believed that DXR has the ability to generate sufficient ROS to raise oxidative stress. Indeed, we observed DXR-induced ROS generation in both CD44^+^CD133^−^ and CD44^+^CD133^+^ cells, but the DXR-induced ROS generation was detected even higher in CD44^+^CD133^−^ than CD44^+^CD133^+^ cells, independent on the intracellular accumulation of DXR (Figures 5(a), 2(b), and 3(b)), suggesting the enhanced antioxidant capacity in CD44^+^CD133^+^ cells.

The Keap1-Nrf2 control system plays a central role in the antioxidant defense mechanisms. Nrf2 is known as a transcription factor to activate various genes involving in biological defense mechanisms. It has been reported that Nrf2 is constantly expressed in many cancer cells [31–36]. Moreover, the enhanced expression of Nrf2 has been confirmed to associate with poor prognosis of cancer patients [37–41]. In our study, Nrf2 expression was detected higher in CD44^+^CD133^+^ than CD44^+^CD133^−^ cells, and the difference in Nrf2 expression was observed even clearer between cells with DXR administration, independent on the DXR accumulation (Figure 5(b)). These findings also clearly indicate the enhanced antioxidant capacity in CD44^+^CD133^+^ cells. Although the absence of direct evidence by interference experiment, pathways involving in the stress protection and redox regulation might at least partially contributed to the DXR resistance of CD44^+^CD133^+^ cells.

Very strangely, our data showed that the silencing of CD133 expression in CD44^+^CD133^+^ cells by siRNA could only partially increase the cytotoxicity of DXR (Figure 6(b)) but did not change the expression of ABCB1 and the intracellular accumulation of DXR (Figure 6(c)). Other unknown mechanisms beyond the drug excretion and redox regulation are asked to be defined on the DXR resistance of CD44^+^CD133^+^ cells.

Based on data from the present study, the expression of CD133, rather than CD44, more closely associated with the resistance of cancer cells to anticancer drug. As complex mechanisms, including the drug excretion and redox regulation, are likely involved in the drug resistance of CSCs, multiple approaches may be needed to overcome the big problem of drug resistance in cancer patients.

## Data Availability

The data that support the findings of this study are available from the corresponding author upon reasonable request.

## Abbreviations

ABCB1: ATP-binding cassette subfamily B member 1/P-glycoprotein/multidrug resistance protein 1/MDR1
ABCG2: ATP-binding cassette subfamily G member 2/breast cancer resistance protein/BCRP/CD388
ABCC1: ATP-binding cassette subfamily C member 1/multidrug resistance-associated protein 1/MRP1
BSO: Buthionine sulfoximine
CSCs: Cancer stem cells
DXR: Doxorubicin/Adriamycin
MTT: 3-(4,5-Dimethylthiazol-2-yl)-2,5-diphenyltetrazolium bromide
Nrf2: Nuclear factor erythroid 2-related factor 2
p38MAPK: p38 MAP kinase
ROS: Reactive oxygen species
siRNA: Small interfering RNA.
